# Validation of a dual-task exercise program to improve balance and gait speed in older people (DualPro): a Delphi study

**DOI:** 10.7717/peerj.13204

**Published:** 2022-04-05

**Authors:** Luz Adriana Varela-Vásquez, Montserrat Girabent-Farrés, Almudena Medina-Rincón, Sandra Rierola-Fochs, Javier Jerez-Roig, Eduard Minobes-Molina

**Affiliations:** 1 Research Group on Methodology, Methods, Models and Outcomes of Health and Social Sciences (M_3_O), Faculty of Health Sciences and Welfare, University of Vic-Central University of Catalonia (Uvic-UCC), Vic, Barcelona, Spain; 2 Department of Physiotherapy, School of Health Sciences, TecnoCampus-Pompeu Fabra University, Mataró, Barcelona, Spain; 3 RE-FIT Barcelona Research Group, Parc Sanitari Pere Virgili and Vall D’Hebrón Research Institute (VIHR), Barcelona, Spain

**Keywords:** Dual-task, Delphi method, Balance, Gait speed, Therapeutic exercise, Older adult

## Abstract

**Background:**

Most physical exercise programs for older people work the physical component in isolation, excluding cognitive aspects. Previous studies reported that both components (physical and cognitive) are necessary for correct functioning of older people in the society.

**Purpose:**

To create and validate a dual-task exercise program (DualPro) to improve balance and gait speed in older people.

**Methods:**

Expert consensus or the Delphi Method was used for validation. A group of 17 experts in neurorehabilitation and geriatrics was recruited to assess the proposed exercise program. They were selected taking into account their experience in clinical practice as well as their knowledge of the subject through the use of the expert competence coefficient (K). Online questionnaires were sent with a total of 11 exercises, which had to be rated using a “Likert” scale from 1 to 7.

**Results:**

Two rounds were conducted to achieve 100% consensus in all exercises. The interquartile range of each exercise in both rounds was stable. During the second round, the relative interquartile range was less than 15% in all the questions, thus demonstrating consensus among the experts.

**Conclusion:**

Experts in neurorehabilitation and geriatrics have concluded the validity of the progressive and systematized program of dual-task exercises focused on improving balance and gait speed for older people. This exercise program can help in the homogenization of the use of dual-task exercises in future studies and in professional practice.

## Introduction

The World Health Organization indicates that the population over 60 will double from 12% to 22% in the period between 2015 and 2050. It has been estimated that by 2050 there will be 434 million people over 80 in the world (World Health Organization, 2016). Population aging and its consequences at the political, economic, social, and health levels are nowadays the subject of multiple investigations, which seek to offer a framework to guarantee attention to the needs and rights of the older people (World Health Organization, 2016). In the context of these investigations, health professionals have to face the challenge of generating treatment strategies through physical exercises that prolongs not only life (Chattejl et al., 2015; World Health Organization, 2012), but also good health in added years of life, thus contributing to healthy aging that seeks proper physical and cognitive functioning through active aging (Sociedad Española de Geriatría y Gerontología (SEGG), 2014).

Among common motor alterations in aging patients are a decrease in range of motion, loss of strength and muscle weakness, which in turn influence posture maintenance and postural control, thus also influencing the balance and gait of older people (Islam et al., 2018; Clancy et al., 2015). In addition to motor deficits, there are cognitive alterations at the attention level, executive functions and memory that can affect movement planning, generating problems in the execution, modification and adaptation of movement to the demands of the environment. These alterations are common in older people, generating problems in the integration of motor and cognitive information necessary for functional actions that imply balance and gait maintenance (Casas-Herrero & Montero-Odasso, 2010; Mazaheri et al., 2014). Belonging to a population group whose deficits are not only motor but also cognitive in nature makes alterations of balance and gait, and the risk of falls, more evident and, therefore, more necessary to treat (Islam et al., 2018).

Evidence that interventions focused on physical exercise are effective in delaying and even reversing losses of strength, muscle mass, flexibility and other physical conditions (Clegg, 2014) shows us the importance of implementing physical treatment tools that can be established in the community. Currently, there are physical exercise programs designed to reduce the risk of falls (Campbell & Robertson, 2003), as well as to prevent and improve frailty (Izquierdo et al., 2017). For the most part, these programs usually include types of physical exercises such as strength, flexibility, the aerobic component and balance, and, to a lesser extent or not at all, the cognitive training and dual-task (DT) processing.

The dual-task is defined as the performance of simultaneous form of two activities that can be motor or cognitive-motor, which generate the integration of multiple brain processing systems, where cognitive funtions play an important role (Enríquez-Reyna et al., 2013; Falbo et al., 2016; McLeod, 1977). This processing capacity of two activities is diminished in older people (Watanabe & Funahashi, 2014), so their training acquires a relevant role and therefore the incorporation of DT as a training tool (Bherer, 2015), Currently there is evidence that both components (physical and cognitive) are necessary for a correct functioning of people in society, since it is the usual form of functioning in the daily life of human beings (Azadian et al., 2016; Gregory et al. 2016; Silsupadol et al., 2009).

The DT exercise method requires the performance of exercises that are more complex than the exercises of the usual programs for older people, since consists of two tasks that are performed simultaneously, generating greater brain activation to be able to carry them out (Silsupadol et al., 2006). The tasks that comprise a DT exercise can be both motor tasks (motor-motor DT) or one cognitive and one motor task (cognitive-motor DT); the training of both combinations is important for DT function (Silsupadol et al., 2006).

This exercise method may vary depending on the instructions given to the individual with fixed prioritization DT training, when the individual is asked to place the same level of attention on both tasks, and variable prioritization DT training, when the individual is asked to shift focus from one task to the other (Kramer, Larish & Strayer, 1995).

In line with the importance of carrying out a physical exercise program for this population that includes cognitive training, there is evidence that this type of treatment improves gait speed in community-dwelling older people without dementia (Gregory et al. 2016), and also improves balance and represents a fall prevention strategy in older people with balance disorders (Silsupadol et al., 2009). Furthermore, there is evidence suggesting that DT exercises may improve cognition in people at risk of cognitive impairment (Bherer, 2015).

The practice of simultaneous tasks is indispensable for task coordination skills and cognitive and motor performance (Kramer, Larish & Strayer, 1995; Shumway-Cook & Woollacott, 2012; Springer et al., 2006). Evidence indicates that DT exercises should be planned to meet certain characteristics such as including the use of different DT training modalities and training of specific concepts, which is crucial in motor learning (Kramer, Larish & Strayer, 1995; Lemke et al., 2019). At the same time, it is pointed out that the training of functional activities in a single task may not be transferable to the performance of the same functional activities in DT (Silsupadol et al., 2009).

Despite the evidence supporting the importance of addressing physical function through DT to improve or maintain the balance and gait speed in older people, today there is no protocolized and progressive exercise program that addresses aspects such as balance and gait using DT. Therefore, the objective of this study is to develop and validate a DT program for balance and gait training (DualPro) in the older people, based on exercises that have previously been independently shown to be effective (Varela-Vásquez, Minobes-Molina & Jerez-Roig, 2020), including both physical and cognitive functions as a necessary tool to enhance the possibilities of active and healthy aging.

## Materials & Methods

The present study consists of the design of a DT exercise program to improve balance and gait speed for older people, using the Delphi method. The Delphi method procedure involves completing a questionnaire on a specific field, in which a panel of experts evaluates the adequacy of the program. A series of rounds are established until consensus is reached among the experts. In each one of the rounds after the first, the experts reevaluate their opinion based on the grouping of responses obtained in the previous round (Varela-Ruiz, Díaz-Bravo & García-Durán, 2012). It is an anonymous procedure in which the experts do not know the identity of the other members of the expert panel who will answer the questionnaires. It is also is an interactive and repetitive procedure, which ends with the consensus of all the experts on the proposed topic (Landeta, 2006).

### Researcher characteristics

The research team consisted of five physiotherapists (LV, AM, SR, JJ and EM), all university professors in areas related to neurorehabilitation, geriatrics and community health. The sixth member of the research team (MG) is an expert in statistics and research methodology, with extensive experience in applied research in physiotherapy.

The steering committee is made up of three members (LV, JJ and EM) who designed and analyzed the responses and comments, where the role of moderator was carried out by the principal investigator (LV). In addition, an advisory team was established, made up of three people external to the study (MG, AM and SR), in order to ensure the correct methodological process of the Delphi study.

### Exercise program design

Based on the scientific evidence found through a bibliographic review, we designed a DT exercise program focused on balance and gait speed for older people (Varela-Vásquez, Minobes-Molina & Jerez-Roig, 2020). Subsequently, the validation of the program was performed using the Delphi method to reach an expert consensus.

### Participants

After selection criteria, LV (principal investigator) was in charge of contacting health professionals of Spain and South America. Seventeen experts were identified who met the criteria and were easily accessible by email. The experts were physical therapists, occupational therapists or rehabilitation doctors, with more than five years of clinical experience in neurorehabilitation or geriatrics.

An email was sent with the information of the project and requesting collaboration in the Delphi process. In this email, they were asked to participate in the study, giving them the information on the study, and if they were interested, they were asked to respond to four items to self-assess their expert level. This allowed us to calculate the coefficient of expert competence (K), which is defined as the mean of the knowledge coefficient (Kc) and the argumentation coefficient (Ka), and enabled us to identify the experts, taking into account different characteristics. These characteristics are of a formative, professional and personal nature, and help to classify an individual as an expert in a certain subject (Cabero-Almenara & Barroso-Osuna, 2013; Cabero-Almenara & Infante-Moro, 2014).

The expert coefficient (K) was performed using four questions to determine Kc and Ka. In the first question they had to value from 0 to 10 their level of knowledge in the subjects that intervene in the study (Kc). In the following three, they had to value as low, medium or high the influence of factors defined by the coordinating group, as well as the weight established for each factor, as recommended by the previous literature (Cabero-Almenara & Barroso-Osuna, 2013; García-Ruiz & Lena-Acebo, 2018). These factors were aspects such as experience obtained in clinical practice (Exp), knowledge of national and international scientific evidence on the subject (Kno), and intuition on the subject and knowledge of technological tools available in the field (Int). These questions constitute the Ka value. After assessing the influence as high for all factors, the weights considered were 0.5 for “Exp”, 0.4 for “Kno”, and 0.1 for “Int”.

\begin{eqnarray*}{k}_{a}= \frac{Exp\ast 0.5+Kno\ast 0.4+Int\ast 0.1}{0.5+0.4+0.1} \end{eqnarray*}

*Equation 1: equation for the calculation of the argumentation coefficient.*

\begin{eqnarray*}K= \frac{{K}_{a}+{K}_{c}}{2} \end{eqnarray*}

*Equation 2: equation for the calculation of the coefficient of expert competence.*

Only those experts whose K coefficient was 0.8 or higher were considered (Cabero-Almenara & Barroso-Osuna, 2013).

### Ethical considerations

This study follows the ethical considerations of the Declaration of Helsinki and data protection regulations in force in Spain. All experts were informed of the objective and procedures of the study, giving their consent to participate online.

The study was evaluated by the Research Ethics Committee of the University of Vic - Central University of Catalonia and approved pursuant to code 88/2019, date June 4, 2019.

### Survey process

An initial questionnaire was prepared and sent *via* email to those professionals who, by calculating the expert coefficient, determined themselves to be experts in the subject in question. This questionnaire consisted of 11 questions, in which the expert was asked to assess the adequacy of each of the exercises proposed in the program. In each question, the exercise was explained, and an illustration was shown to support the explanation.

A period of three weeks was given to answer each questionnaire, and a reminder was sent a week before the deadline. After receiving the answers to the first questionnaire, they were analyzed, and based on the results, the second questionnaire described below was generated. This second questionnaire had to be answered taking into account the group of previous answers. This process was repeated as many times as necessary until consensus was reached.

The questionnaire evaluates separately the exercises that constitute the DT program (DualPro) to improve balance and gait speed of the older people. The proposed instrument consists of 11 exercises, 3 motor-motor DT exercises (DMT), and 8 cognitive-motor DT exercises (DCMT), divided into five levels that progressively increase in difficulty. This progression is given by the requirement of the tasks included in each exercise and by the level of attention that is requested at each level. The motor tasks included were manipulative, maintaining balance and gait in different conditions of support surface, whereas the cognitive tasks were tasks of verbal fluency, discrimination and decision-making, memory, mental monitoring and reaction time. These tasks address cognitive functions of memory, attention, and executive functions.

The DualPro program is divided as follows: the first level consists of a sitting DT exercise; the second level consists of 3 balance exercises in DT where the support base and the secondary task vary; the third level consists of 2 gait exercises in motor-motor DT; the fourth level includes 3 cognitive-motor DT gait exercises and the fifth level 2 gait exercises that seek to transfer DT to daily activities.

The program begins with three levels of fixed prioritization, whereby attention is shared equally between both tasks, and progresses at levels four and five to variable prioritization training, in which the proportion of attention directed to walking and the added task may vary according to the physical therapist’s instructions. For progress at each level of the DualPro exercise program, the older people must be able to perform the exercises in the previous level safely, maintaining the performance of the balance or gait exercise in DT for a time in seconds that has been established for each exercise. During the performance of balance exercises in DT, the individual should not present significant imbalances that force him to grab an object or stop performing the added task. In walking DT exercises, the participant must perform them without stopping to stabilize himself.

The expert evaluated each exercise according to the clarity, suitability for the objective, suitable for older people with mild to moderate balance and gait limitations, as well as cognitive impairment. The expert also had to consider the level of difficulty and progression of each exercise within the program. This assessment was carried out globally using a 7-point Likert scale, from 1 to 7 (“Totally disagree” =1; “Strongly disagree” =2; “Disagree” =3; “Neither agree nor disagree” =4; “Agree” =5; “Strongly agree” =6; “Totally agree” =7). In addition, a blank space was attached so that they could suggest modifications or give their opinion on the weak and strong points of the exercise.

In the first round, experts had the option to respond using a 7-point Likert scale, categorizing and ordering their answers according to the degree of agreement, the results of which became the starting point for subsequent opinions (Reguant-Álvarez & Torrado-Fonseca, 2016). Due to this, after the first round, the experts were informed of the results obtained in the previous round through a graph that showed the responses and the comments expressed by the experts. The anonymity of the expert was maintained at all times so that subsequent answers were not influenced (López-Gómez, 2018).

### Statistical analysis

The data obtained in each round were analyzed in order to establish consensus. A record of each of the responses obtained in each round was made in version 16.55 of the Excel database, in order to allow the calculation of minimum value, maximum value, mean, standard deviation (SD), quartile 1 (Q1), the median (Me), quartile 3 (Q3) and the interquartile range (IQR), as well as the table of absolute and relative frequencies.

On the basis of the aforementioned statistical indices, it was considered that there was consensus in each of the questions if there was convergence between the values of Q1 and Q3; that is, whenever IQR (IQR = Q3−Q1) tends to 0 or if the Relative Interquartile Range (RIR = (Q3−Q1) * 100/Me) was less than 15% (Rayens & Hahn, 2000; Novakowski & Wellar, 2008).

This analysis was carried out for each round. If there was no consensus, the new questionnaire was generated, which was sent along with a graph per question, where the behavior of the responses in the previous round was presented and they were asked to reassess. The experts were not identified with the answers so that anonymity among them was guaranteed.

In addition to the previous analysis, the Fleiss Kappa coefficient (g) was calculated in order to assess the degree of agreement (reliability) between the experts’ responses. The interpretation of this coefficient was based on the following criteria (Fleiss, Levin & Paik, 2013): (a) g ≤0.4: weak or poor reliability; (b) 0.4 < g ≤ 0.6: moderate reliability; (c) 0.6 < g ≤ 0.8: good reliability and, (d) g > 0.8: excellent reliability.

## Results

### Expert panel

The final group of experts assessed their level of expertise in the subject with the knowledge coefficient (kc) whose mean was 0.85 (SD 0.09), and their argumentation coefficient level (ka) 0.95 (SD 0.07). Likewise, the average of the expert competence coefficient (K) was 0.90 (SD 0.07). Seventeen professionals were initially identified as potential experts, after calculating the K coefficient the final number of volunteers included as experts was fourteen. Three experts were excluded for not getting a score whose K coefficient was 0.8 or higher (Cabero-Almenara & Barroso-Osuna, 2013; Table 1). The level of expertise of the members of the expert panel was at the threshold considered high.

**Table 1:** Calculation of the expert competence coefficient (K).

**Expert** **code**	**Self valuation**	**kc**	**ka**	**K**
E1	8	0.80	0.80	0.80
E2	8	0.80	0.87	0.83
E3	8	0.80	0.87	0.83
E4	10	1.00	1.00	1.00
E5	8	0.80	0.97	0.88
E6	9	0.90	1.00	0.95
E7	8	0.80	0.97	0.88
E8	7	0.70	0.97	0.83
E9	9	0.90	1.00	0.95
E10	8	0.80	1.00	0.90
E11	9	0.90	1.00	0.95
E12	8	0.80	0.83	0.82
E13	9	0.90	0.97	0.93
E14	10	1.00	1.00	1.00
E15^*^	7	0.70	0.67	0.68
E16^*^	5	0.50	0.67	0.58
E17^*^	7	0.70	0.80	0.75

**Notes.**

kc Knowledge coefficient ka Argumentation coefficient K The expert competence coefficient

* Excluded experts.

### Validation

The percentage of participation in the validation of the program was 100% (*n* = 14) in the first and second round. Only two rounds were necessary to validate the designed DT exercise program to improve balance and gait speed for older people. In the first round, the participants answered, “Totally agree” (7 points) or “Strongly agree” (6 points) in 5 (45.45%) of the 11 exercises proposed. In these 5 exercises, the percentage of “Totally agree” was greater than 60%. In general, we can observe that none of the exercises received a rating of disagreement.

During the first round, the questions referring to the DT-seated knee raise, DMT-on unstable surface, DMT-lateral gait and DCMT-backward gait exercises were the ones that generated the greatest discrepancy. Similarly, during this round, some experts suggested modifications to some exercises, which were as follows: for the DT-seated knee raise exercise, they recommended taking into account hip pain and impediments in flexion greater than 90 degrees in the case of hip joint replacements; in the DMT-on unstable surface and DCMT- on unstable surface exercises, a common suggestion was to define the type of unstable surface, since there are different types in the rehabilitation gyms, most recommending the foam balance pads. For the rest of the exercises, the most common recommendation was to define the duration of each exercise, between 30 and 60 s.

**Figure 1:**
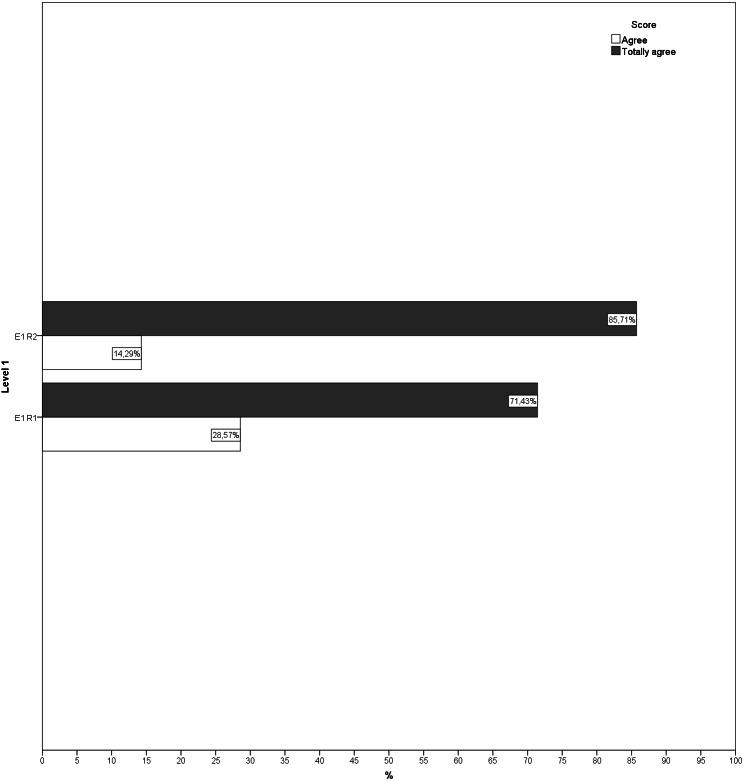
Answers to questions regarding level 1 in both rounds. E1, Exercise 1; R1, Round; R2, Round 2.

**Figure 2:**
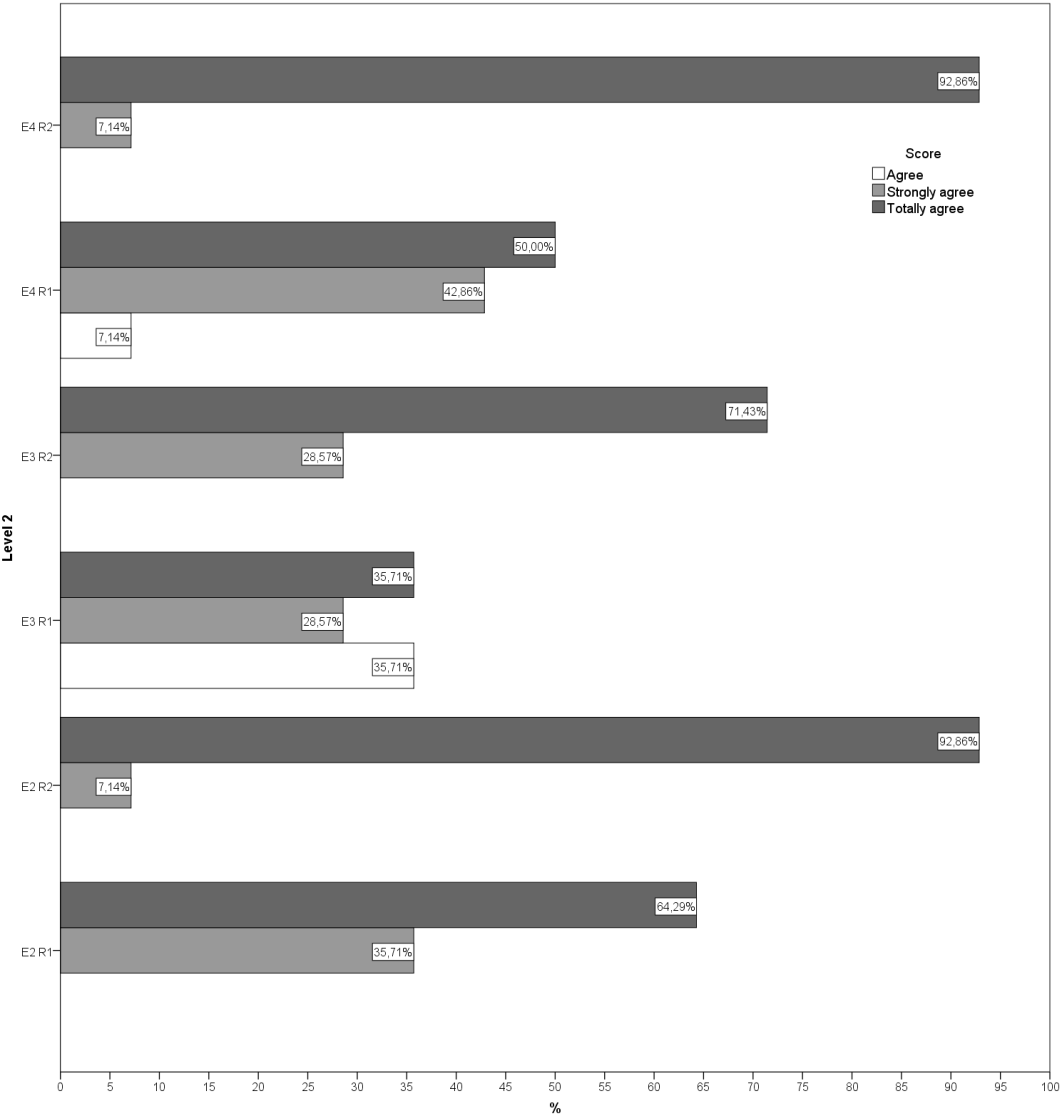
Answers to questions regarding level 2 in both rounds. E2, Exercise 2; E3, Exercise 3; E4, Exercise 4; R1, Round; R2, Round 2.

**Figure 3:**
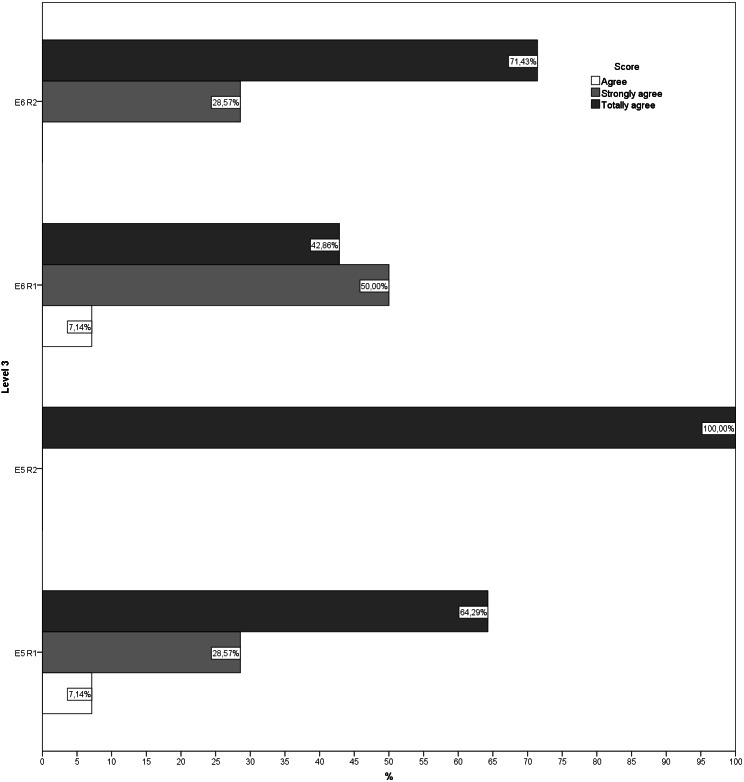
Answers to questions regarding level 3 in both rounds. E5, Exercise 5; E6, Exercise 6; R1, Round; R2, Round 2.

**Figure 4:**
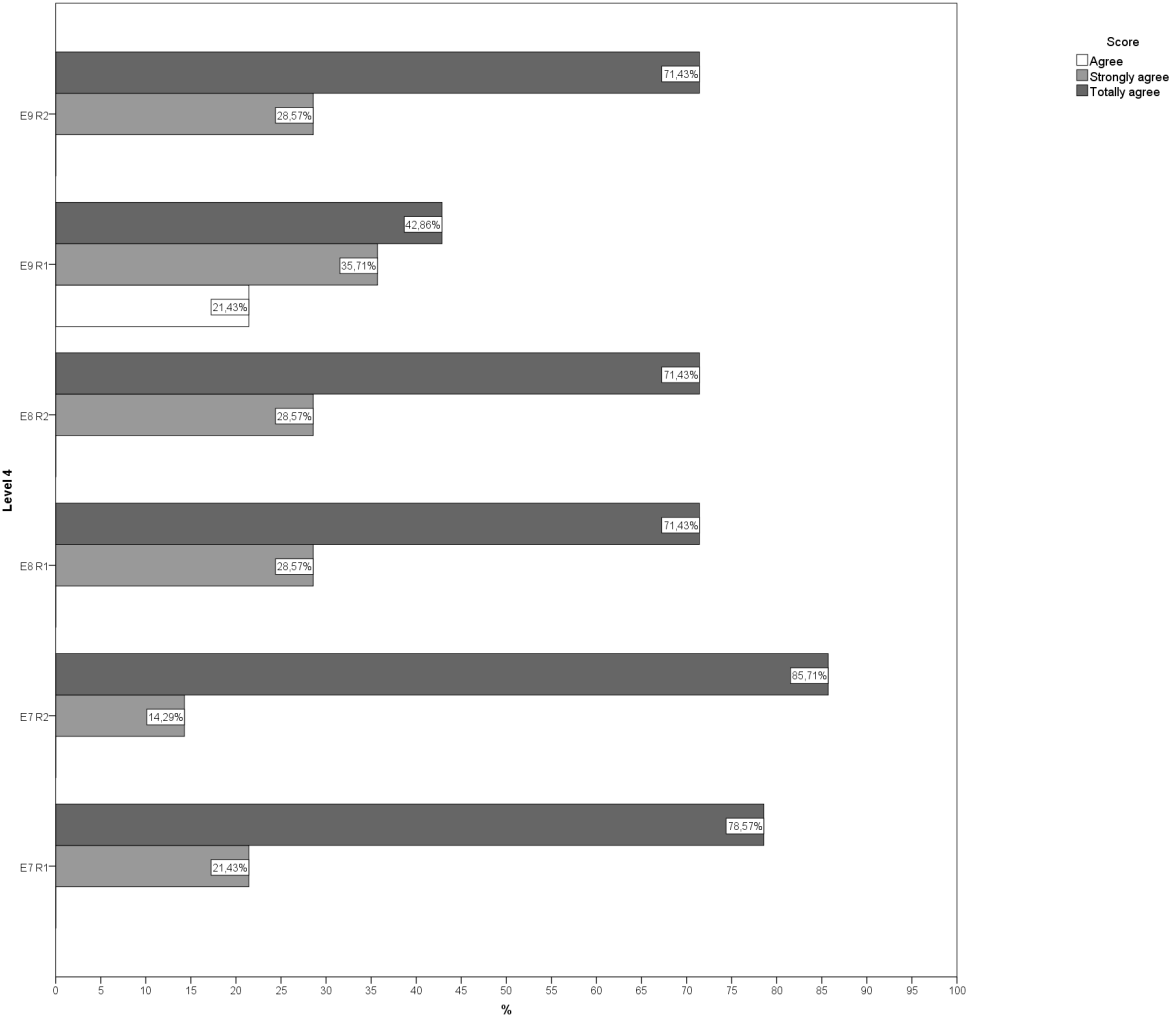
Answers to questions regarding level 4 in both rounds. E7, Exercise 7; E8, Exercise 8; E9, Exercise 9; R1, Round; R2, Round 2.

**Figure 5:**
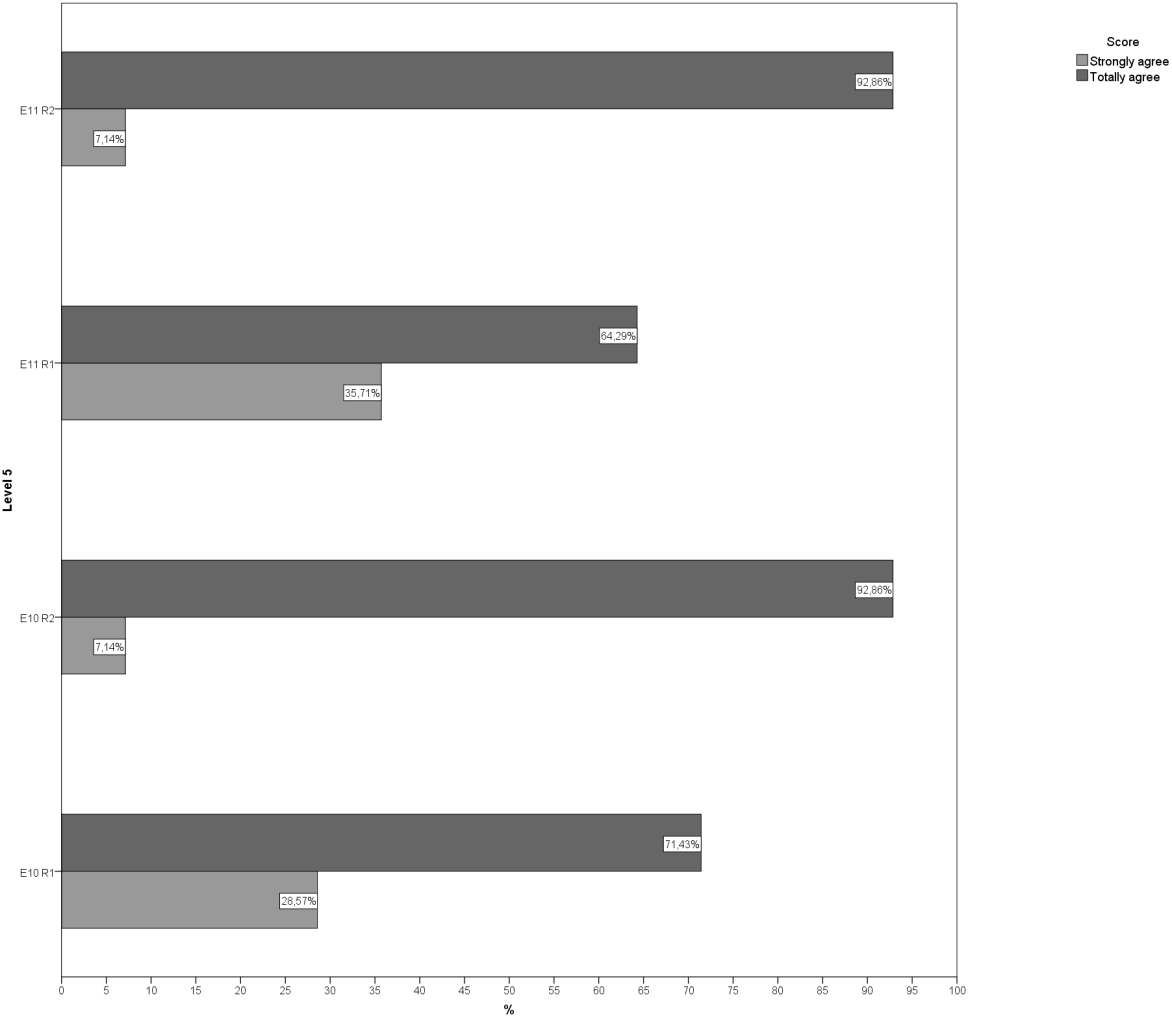
Answers to questions regarding level 5 in both rounds. E10, Exercise 10; E11, Exercise 11; R1, Round; R2, Round 2.

During the second round, more than 70% of the answers given by the experts for each question showed that they were in agreement, indicating that there was little dispersion in the answers. Likewise, no new suggestions for changes or improvements were received for any of the exercises (Figs. 1–5). The IQR of each exercise in both rounds remained stable (the DCMT-gait exercise resulted in IQR = 0 and the DCMT-lateral gait exercise in IQR = 0.75, in both rounds) and decreased in the rest of the exercises. In addition, during the second round the RIR was less than 15% for all the questions, indicating that agreement had been reached among the experts in each of the exercises that make up the DualPro program (Table 2, Appendix 1 *(full program)*).

The Fleiss Kappa index for multiple observers showed a degree of agreement between the experts of 0.720, with a confidence interval (CI) of 95% of [0.440, 0.894] in the first round, which is a good concordance. In the second round, degree of agreement was 0.848 with a 95% CI of [0.696, 0.943], which is an excellent degree of agreement.

## Discussion

In this study, using the Delphi method, it has been possible to create and validate a DT exercise program for older people that may improve balance and gait training (DualPro). In addition, it can complement other exercise programs for older people. These programs usually take into account specific exercises such as strength, flexibility, cardiovascular exercise or balance, but frequently do not include DT training (Campbell & Robertson, 2003). Occasionally, they contemplate DT exercises related to the specific balance function (Izquierdo et al., 2017). However, the validated program contemplates the use of DT exercise as fundamental, since it is the usual form of functioning in the daily life of human beings (Azadian et al., 2016).

**Table 2:** Dual-task exercise program to improve balance and gait speed in older people, resulting from consensus. DualPro

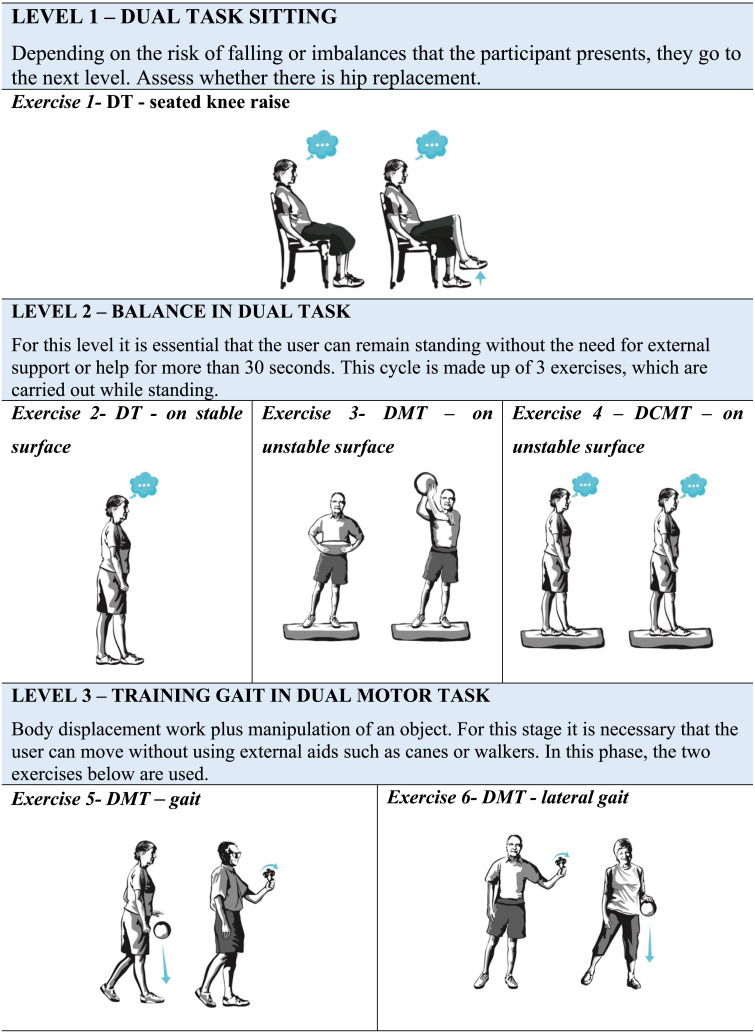
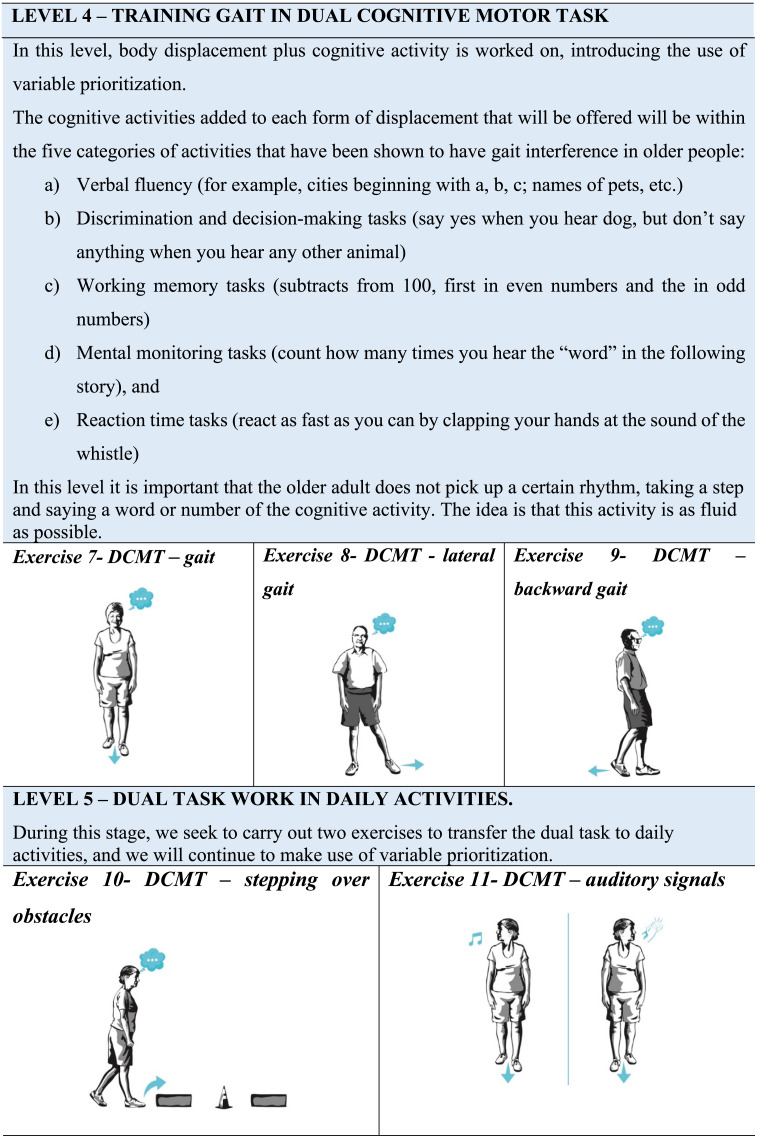

**Notes.**

DT, dual task; DMT, dual task motor-motor; DCMT, dual task cognitive-motor.

The DualPro program was created taking in consideration all the reported aspects of DT exercises that seem to be more effective, includes the use of the different DT modalities, with both motor-motor and cognitive-motor dual tasking (Varela-Vásquez, Minobes-Molina & Jerez-Roig, 2020). These tasks start with balance in DT while seated, then progress to static balance in more demanding postures, and adding a second cognitive or motor activity, and finally move on to dynamic balance work that is more demanding in DT.

In addition, prioritization has been considered as part of the training modalities, which can be fixed and variable prioritization. It has been reported that the prioritization modality seems to have an influence on the transfer of training to everyday actions, with greater effects found with variable prioritization (Varela-Vásquez, Minobes-Molina & Jerez-Roig, 2020). Since it is difficult to start with variable prioritization in some older people, a logical sequence of progressive motor learning has been established (Kramer, Larish & Strayer, 1995; Lemke et al., 2019).

The practice of two simultaneous tasks is reported as being essential to improve task coordination activities (Shumway-Cook & Woollacott, 2012), in which cognitive functions are directly involved in the planning and execution of any type of action. It is known that the maintenance of balance and gait are two actions of special importance in the functional independence of older people, as well as the involvement of cognitive functions such as attention, working memory or executive functions (Casas-Herrero & Montero-Odasso, 2010; Gregory et al. 2016; Mazaheri et al., 2014). The importance of the subject of this study is reflected in the existence of multiple studies dedicated to investigating DT strategies as tools to improve, maintain or regain balance and gait capacity (Smith-Ray et al., 2015; Springer et al., 2006).

Regarding balance, improvements have been reported with DT exercise training (Bharti, 2014; Silsupadol et al., 2009; Booth, Hood & Kearney, 2016), and in turn it is suggested that the type of attention focus that is requested appears to be important in DT training, showing that variable prioritization has advantages over fixed prioritization, in terms of transfer aspects (Kramer, Larish & Strayer, 1995; Lemke et al., 2019). It is also suggested that only precise training might improve gait performance in DT; and that training in a single task condition might not be enough to improve gait in the DT condition, a capacity that is considered necessary for a functional gait (Silsupadol et al., 2009).

Gait speed is considered to be an indicator of functional balance and risk of falling in older people (Cesari et al., 2005; Hardy, et al., 2007). An improvement of 0.10 m/s in the gait speed of a single task in the older people is considered a substantial change (Hardy, et al., 2007). The exercises included in the DualPro program come from different studies that included DT intervention groups and control groups that mostly performed different types of exercises without DT, changes were reported indicating that both the participants who performed DT and those who did not, obtained changes in gait speed that exceed the value of 0.10 m/s (Varela-Vásquez, Minobes-Molina & Jerez-Roig, 2020). However, the results of DT training are superior to single task training in terms of balance and gait speed in both single and DT conditions (Varela-Vásquez, Minobes-Molina & Jerez-Roig, 2020).

Studies report that DT training exercises have clinically important implications, both in the cognitive and motor components. The main ones are at the motor level, with repercussions in balance and gait (Smith-Ray et al., 2015; Springer et al., 2006) and at a cognitive level, improvements are reported in the cognitive efficacy of older people and in their executive functions (Gregory et al. 2016; Silsupadol et al., 2009). According to the evidence, application of DT seems to be a promising line of training in older people, provided that it is planned taking into account the minimum necessary cognitive requirements, the use of the type of prioritization, and the training of the activities in which it is intended to have transfer (Watanabe & Funahashi, 2014; Lemke et al., 2019; Ho & Scialfa, 2002).

Regarding the limitations of the study, it is not yet possible to speak of effectiveness since its clinical applicability has not been proven. To determine that a protocol is effective, expert validation is a necessary part of the clinical trial. In addition, the difference in specialization of the group of experts in neurology and geriatrics could have constituted the main disagreements in the first round. This was noted in some of the comments received by the moderator during the Delphi rounds process. However, this diversity may, in turn, be one of the study’s strengths because of the knowledge of both the experts in neurorehabilitation and balance treatment and the experts in the geriatrics, who provide a broader and more specific view of the study population for which the validated exercise program is intended.

It is important to highlight that this program has been validated in Spanish, and that all the participants in the expert group are native Spanish speakers. As a result, there is no issue of linguistic misunderstanding with regards to the questions. On the other hand, the use of a 7-point Likert scale represents a strength, since it shows better internal consistency in this type of study than the 5-point Likert scales (Matas, 2018; González-Alonso & Pazmiño-Santacruz, 2015).

In relation to the validation through the Delphi method, it should be pointed out that this method is widely used in other disciplines such as education (Cabero-Almenara & Infante-Moro, 2014), medical sciences and nursing (Varela-Ruiz, Díaz-Bravo & García-Durán, 2012). In the field of physiotherapy, the consensus of experts using the Delphi method has been less applied, although in the last decade some references can be found in the discipline (Maissan et al., 2018; Van der Lee, Hill, & Patman, 2019; Orhan et al., 2019).

The Delphi method gave us the opportunity to consider information that was not initially included in the studies we took the exercises from, information that was included in the description of the exercise as recommendations or indications to take into account in the application of the program in older people, guidelines on the specific exercise of balance, on specific times or health conditions to monitor in specific exercises. It also showed a convergence on the opinion of the experts on the selected exercises and the order in which they were established, reaching an agreement in the second round.

As far as we know, this is the first expert consensus, generated through a reported Delphi method, on specific DT exercises as being essential in physical exercise programs for older people, addressing aspects such as balance and gait using DT.

## Conclusions

The DualPro exercise program for improving balance and gait speed in older people is valid according to expert consensus using the Delphi method. It could be expected that this program, added to multi-component training programs and exercise plans followed by older people, will enhance the gains in balance and gait speed, delaying the appearance of risk and fear of falling. Studies are needed to evaluate the effectiveness and suitability of the program.

## Supplemental Information

10.7717/peerj.13204/supp-1 Supplemental Information 1 Dual-task exercise program to improve balance and gait speed in older people, resulting from consensus

10.7717/peerj.13204/supp-2 Supplemental Information 2 Delphi database

10.7717/peerj.13204/supp-3 Supplemental Information 3 Questionnaire for the assessment of the dual-task exercise program model for the improvement of balance and gait speed in the elderly

## Additional Information and Declarations

### Data Availability

The following information was supplied regarding data availability: The raw data are available in the Supplementary Files.

## References

[ref-1] Azadian E, Torbati HRT, Kakhki ARS, Farahpour N (2016). The effect of dual task and executive training on pattern of gait in older adults with balance impairment: a randomized controlled trial. Archives of Gerontology and Geriatrics.

[ref-2] Bharti C (2014). Effect of training balance under dual task with fixed and variable priority instructions with balance impairment in institutionalized elderly population. The Indian Journal of Physiotherapy & Occupational Therapy.

[ref-3] Bherer L (2015). Cognitive plasticity in older adults: effects of cognitive training and physical exercise. Annals of the New York Academy of Sciences.

[ref-4] Booth V, Hood V, Kearney F (2016). Interventions incorporating physical and cognitive elements to reduce falls risk in cognitively impaired older adults: a systematic review. JBI Database of Systematic Reviews and Implementation Reports.

[ref-5] Cabero-Almenara J, Barroso-Osuna J (2013). La utilización del juicio de experto para la evaluación de TIC: el coeficiente de competencia experta. Bordon Revista de Pedagogía.

[ref-6] Cabero-Almenara J, Infante-Moro A (2014). Empleo del método Delphi y su empleo en la investigación en comunicación y educación. Edutec. Revista Electrónica de Tecnología Educativa.

[ref-7] Campbell AJ, Robertson MC (2003). Otago exercise programme to prevent falls in older adults.

[ref-8] Casas-Herrero A, Montero-Odasso M (2010). Trastornos de la marcha y demencia. Avances en demencia. Una perspectiva integral.

[ref-9] Cesari M, Kritchevsky S, Pennix B, Nicklas BJ, Simonsick EM, Newman AB, Tylavsky F, Brach J, Satterfield S, Harris T, Pahor M (2005). Prognostic value of usual gait speed in well-functionationing older people-results from the health, ageing and body composition study. Journal of the American Geriatrics Society.

[ref-10] Chattejl S, Byles J, Crutler D, Seeman T, Verdes E (2015). Health, functioning and disability in older adults –current status and future implications. Lancet.

[ref-11] Clancy A, Balteskard B, Perander B, Mahler M (2015). Older persons’ narrations on falls and falling-Stories of courage and endurance. International Journal of Qualitative Studies on Health and Well-Being.

[ref-12] Clegg A (2014). Frailty in older people. The Lancet.

[ref-13] Enríquez-Reyna MC, Cruz-Quevedo JE, Celestino-Soto IM, mGarza Elizondo ME, Salazar-González BC (2013). Función ejecutiva, velocidad de marcha y tarea doble en adultos mayores mexicanos. Revista Iberoamericana de Psicologia del Ejercicio y el Deporte.

[ref-14] Falbo S, Condello G, Capranica L, Forte R, Pese C (2016). Dual task training on executive function and gaiy performance in older adults: a randomized controlled trial. BioMed Research International.

[ref-15] Fleiss JL, Levin B, Paik MC (2013). Statistical methods for rates and proportions.

[ref-16] García-Ruiz ME, Lena-Acebo FJ (2018). Aplicación del metodo delphi en el diseño de una investigación cuantitativa sobre el fenómeno FABLAB. Empiria. Revista de Metodología de Ciencias Sociales.

[ref-17] González-Alonso J, Pazmiño Santacruz M (2015). Cálculo e interpretación del Alfa De Cronbach para el caso de validación de la consistencia interna de un cuestionario, con dos posibles escalas tipo Likert. Revista Publicando.

[ref-18] Gregory MA, Gill DP, Zou G, Liu-Ambrose T, Shigematsu R, Fitzgerald C, Petrella RJ (2016). Group-based exercise combined with dual-task training improves gait but not vascular health in active older adults without dementia. Archives of Gerontology and Geriatrics.

[ref-19] Hardy SE, Perera S, Roumani YF, Chandler JM, Studenski SA (2007). Improvement in usual gait speed predicts better survival in older adults. Journal of the American Geriatrics Society.

[ref-20] Ho G, Scialfa CT (2002). Age, skill transfer, and conjunction search. Journals of Gerontology: Series B.

[ref-21] Islam MS, Mondal MNI, Tareque MI, Rahman MA, Hoque MN, Ahmed MM, Khan HTA (2018). Correlates of healthy life expectancy in low- and lower-middle-income countries. BMC Public Health.

[ref-22] Izquierdo M, Casas-Herrero A, Zambom-Ferraresi F, Marínez-Velilla N, Alonso-Bouzón C, Rodríguez-Mañas L (2017). Programa multicomponente de ejercicio físico para la prevención de fragilidad y riesgo de caídas. Vivifrail, (Erasmus+ 556988-EPP-1-2014-1-ES-SPO-SCP). https://www.munideporte.com/imagenes/documentacion/ficheros/0134414D.pdf.

[ref-23] Kramer AF, Larish JF, Strayer DL (1995). Training for attentional control in dual task settings: a comparison of young and old adults. Journal of Experimental Psychology: Applied.

[ref-24] Landeta J (2006). Current validity of the Delphi method in social sciences. Technological Forecasting and Social Change.

[ref-25] Lemke NC, Werner C, Wiloth S, Oster P, Bauer JM, Hauer K (2019). Transferability and sustainability of motor-cognitive dual-task training in patients with dementia: a randomized controlled trial. Gerontology.

[ref-26] López-Gómez E (2018). El método Delphi en la investigación actual en educación: una revision teórica y metodológica. Educación XXI.

[ref-27] Maissan F, Pool J, Stutterheim E, Wittink H, Ostelo R (2018). Clinical reasoning in unimodal interventions in patients with non-specific neck pain in daily physiotherapy practice, a Delphi study. Musculoskeletal Science and Practice.

[ref-28] Matas A (2018). Diseño del formato de escalas tipo Likert: Un estado de la cuestión. Revista Electronica De Investigacion Educativa.

[ref-29] Mazaheri M, Roerdink M, Bood RJ, Duysens J, Beek PJ, Peper CLE (2014). Attentional costs of visually guided walking: effects of age, executive fuction and stepping-task demands. Gait Posture.

[ref-30] McLeod P (1977). A Dual task response modality effect: suport for multiprocessor models of attention. Quarterly Journal of Experimental Psychology.

[ref-31] Novakowski N, Wellar B (2008). Using the Delphi technique in normative planning research: methodological design considerations. Environment and Planning A: Economy and Space.

[ref-32] Orhan C, Cagnie B, Favoreel A, Van Looveren E, Akel U, Mukhtar NB, Meeus M (2019). Development of culturally sensitive Pain Neuroscience Education for first-generation Turkish patients with chronic pain: a modified Delphi study. Musculoskeletal Science and Practice.

[ref-33] Rayens MK, Hahn EJ (2000). Building consensus using the policy delphi method. Policy, Politics, & Nursing Practice.

[ref-34] Reguant-Álvarez M, Torrado-Fonseca M (2016). El metodo Delphi. REIRE. Revista d’Innovació i Recerca En Educació.

[ref-35] Sociedad Española de Geriatría y Gerontología (SEGG) (2014). Guía de buena práctica clínica en Geriatría.

[ref-36] Shumway-Cook A, Woollacott MH (2012). Motor control: translating research into clinical practice.

[ref-37] Silsupadol P, Lugade V, Shumway-Cook A, Van Donkelaar P, Chou LS, Mayr U, Woollacott MH (2009). Training-related changes in dual-task walking performance of elderly persons with balance impairment: a double-blind, randomized controlled trial. Gait and Posture.

[ref-38] Silsupadol P, Siu K, Shumway-cook A, Woollacott MH (2006). Training of balance under single- and dual-task conditions in older adults with balance impairment. Physical Therapy.

[ref-39] Smith-Ray RL, Hughes SL, Prohaska TR, Little DM, Jurivich DA, Hedeker D (2015). Impact of cognitive training on balance and gait in older adults. Journals of Gerontology - Series B.

[ref-40] Springer S, Giladi N, Peretz C, Yogev G, Simon ES, Hausdorff JM (2006). Dual-tasking effects on gait variability: the role of aging, falls, and executive function. Movement Disorders.

[ref-41] Varela-Ruiz M, Díaz-Bravo L, García-Durán R (2012). Descripción y usos del método Delphi en investigaciones del área de la salud. Investigación en Educación Médica.

[ref-42] Van der Lee L, Hill AM, Patman S (2019). Expert consensus for respiratory physiotherapy management of mechanically ventilated adults with community-acquired pneumonia: a Delphi study. Journal of Evaluation in Clinical Practice.

[ref-43] Varela-Vásquez LA, Minobes-Molina E, Jerez-Roig J (2020). Dual-task exercises in older adults: A structured review of current literature. Journal of Frailty, Scarcopenia & Falls.

[ref-44] Watanabe K, Funahashi S (2014). Neural mechanisms of dual-task interference and cognitive capacity limitation in the prefrontal cortex. Nature Neuroscience.

[ref-45] World Health Organization (2012). La buena salud añade vida a los años. https://www.who.int/world-health-day/2012/global_brief_summary_es.pdf.

[ref-46] World Health Organization (2016). Accion multisectorial para un envejecimiento saludable basado en el ciclo de vida: proyecto de estrategia y plan de acción mundiales sobre el envejecimiento y la salud: Informe de la Secretaría. https://apps.who.int/iris/handle/10665/253025.

