# Supplementary material for: Validation of a dual-task exercise program to improve balance and gait speed in older people (DualPro): a Delphi study
Source: PeerJ. 2022 Apr 5;10:e13204. doi: 10.7717/peerj.13204 (PMC8992645; doi:10.7717/peerj.13204)
Supplement: Supplemental Information 1 [file peerj-10-13204-s001.docx]

***Appendix 1:*** *Dual-task exercise program to improve balance and gait speed in older people, resulting from consensus. DualPro*

| **LEVEL 1 – DUAL TASK SITTING**  Depending on the risk of falling or imbalances that the participant presents, they go to the next level. Assess whether there is hip replacement. |
| --- |
| ***Exercise 1-* DT - seated knee raise**  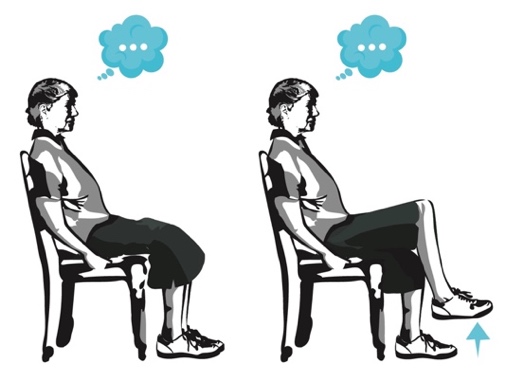  Position: the older adult should be sitting in a standard chair, with a straight back (90º) and correctly supported by the backrest.  Exercise: the user must lift their leg up and down alternating between the right and left leg as quickly as possible. The minimum lifting height will be to lift the plantar surface off the ground. They will be carried out in six series of 15 seconds. Simultaneously with the motor activity, you will be asked for a verbal fluency task, which consists of naming words within a category (example: words that begin with “A”, female names that begin with “M”, names of animals, etc). The verbal fluency task should be changed in each session. |
| **LEVEL 2 – BALANCE IN DUAL TASK**  For this level it is essential that the user can remain standing without the need for external support or help for more than 30 seconds. This cycle is made up of 3 exercises, which are carried out while standing. |
| ***Exercise 2- DT - on stable surface***  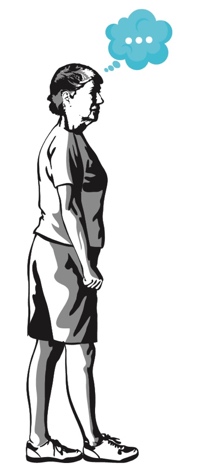  The older adult stands on a firm surface with eyes open, will use a narrow base (feet together) or a tandem posture (depending on the safety demonstrated by the participant) that he/she will have to maintain for 30 seconds while performing a cognitive task that can be, in order of difficulty: randomly name numbers from 100 to 500; association of words by easy categories (animals, fruits, etc); and more difficult associations (names of women beginning with a specific letter, cities, etc).  The difficulty of the cognitive task will depend on the safety of the participant’s standing. There will be two repetitions of 30 seconds. |
| ***Exercise 3- DMT – on unstable surface***  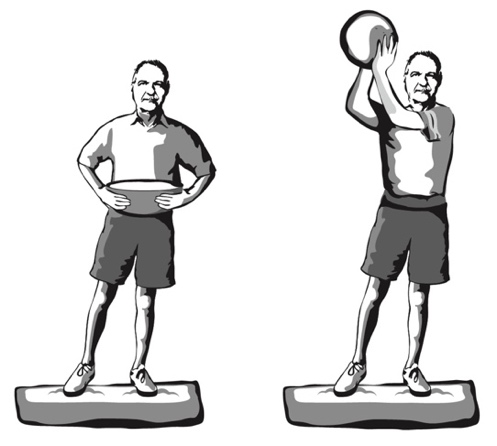  On an unstable surface (yoga pad, foam pad), the subject must manipulate an object such as holding a ball or a container. For greater difficulty the subject must grasp or throw the object. The exercise will be performed for 30 seconds, two repetitions. |
| ***Exercise 4 – DCMT – on unstable surface***  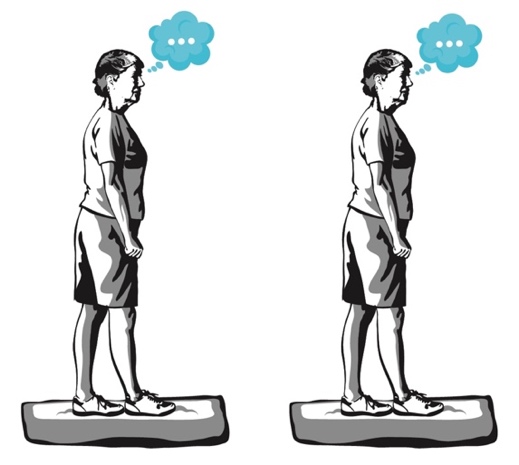  On an unstable foam surface, with eyes open or closed depending on the user’s safety, with a narrow base or tandem posture, you will perform cognitive activities that will consist of randomly naming odd or even numbers in a certain range, word association (easy and more difficult), or spelling short words backwards.  Educational level will be taken into account for cognitive tasks such as spelling or mathematical operations. The exercise will be performed for 30 seconds, two repetitions. |
| **LEVEL 3 – TRAINING GAIT IN DUAL MOTOR TASK**  Body displacement work plus manipulation of an object. For this stage it is necessary that the user can move without using external aids such as canes or walkers. In this phase, the two exercises below are used. |
| ***Exercise 5- DMT - gait.***  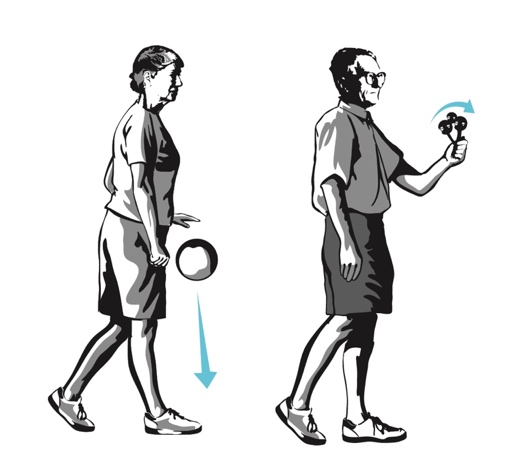  Walk forward, along with a manipulative task, such as walking while raising an umbrella with both hands, while carrying a tray, or while shaking a rattle (less difficulty), walking while bouncing a ball (greater difficulty). The activities with the least difficulty will begin and, depending on the safety of the participant, the ball will be bounced. The exercise will be performed three times, for a period of 30 seconds each time. |
| ***Exercise 6- DMT - lateral gait***  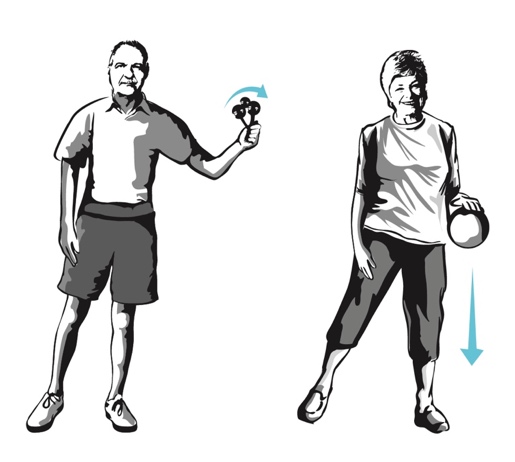  The older adult must make a movement with lateral steps, with manipulative tasks such as those of the previous exercise added to them. The exercise will be performed three times, for a period of 30 seconds each time. |
| **LEVEL 4 – TRAINING GAIT IN DUAL COGNITIVE MOTOR TASK**  In this level, body displacement plus cognitive activity are worked on, introducing the use of variable prioritization.  The cognitive activities added to each form of displacement that will be offered will be within the five categories of activities that have been shown to have gait interference in older people:   1. Verbal fluency (for example, cities beginning with a, b, c; names of pets, etc.) 2. Discrimination and decision-making tasks (say yes when you hear dog, but don’t say anything when you hear any other animal) 3. Working memory tasks (subtracts from 100, first in even numbers and the in odd numbers) 4. Mental monitoring tasks (count how many times you hear the “word” in the following story), and 5. Reaction time tasks (react as fast as you can by clapping your hands at the sound of the whistle)   In this level it is important that the older adult does not pick up a certain rhythm, taking a step and saying a word or number of the cognitive activity. The idea is that this activity is as fluid as possible. |
| ***Exercise 7- DCMT - gait***  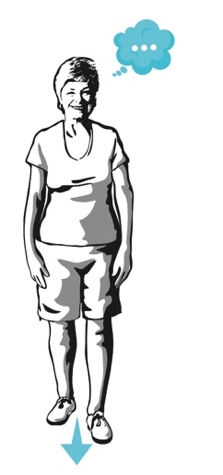  Forward shifts + cognitive activity. The exercise will be performed three times, for a period of 60 seconds each time. |
| ***Exercise 8- DCMT - lateral gait***  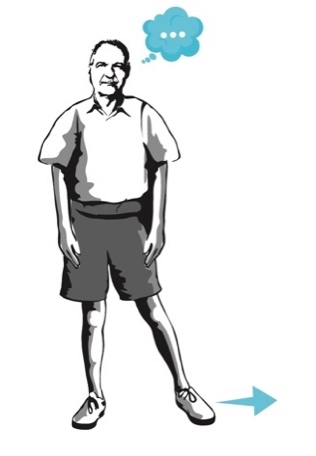  Lateral displacement + cognitive activity. The exercise will be performed three times, for a period of 30 seconds each time. |
| ***Exercise 9- DCMT – backward gait***  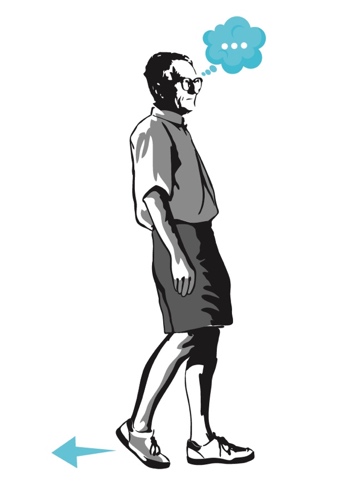  Backward movement + cognitive activity. The exercise will be performed three times, for a period of 30 seconds each time. |
| **LEVEL 5 – DUAL TASK WORK IN DAILY ACTIVITIES.**  During this stage, we seek to carry out two exercises to transfer the dual task to daily activities, and we will continue to make use of variable prioritization. |
| **Exercise 10- DCMT – stepping over obstacles**  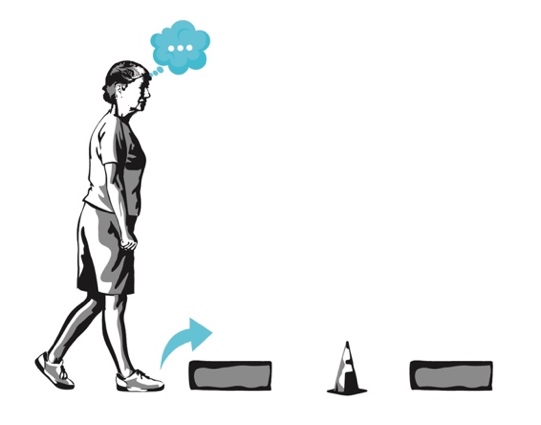  Gait forward avoiding obstacles, while performing a cognitive activity of auditory or visual discrimination. |
| **Exercise 11- DCMT – auditory signals**  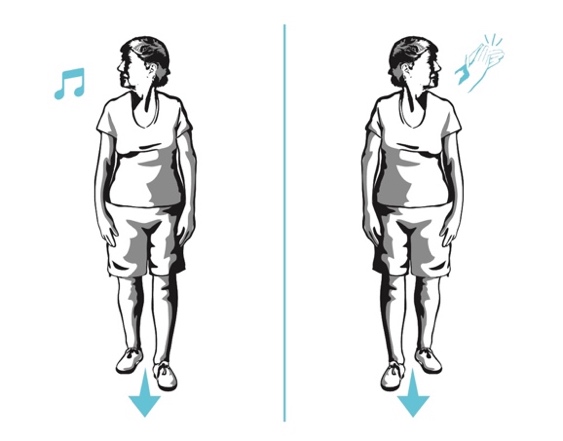  Gait forward, and twist the trunk to one side or the other according to auditory discrimination (when you hear the whistle, turn to the right; when you hear a palm turn to the left). |
