# Supplementary material for: Validation of a dual-task exercise program to improve balance and gait speed in older people (DualPro): a Delphi study
Source: PeerJ. 2022 Apr 5;10:e13204. doi: 10.7717/peerj.13204 (PMC8992645; doi:10.7717/peerj.13204)
Supplement: Supplemental Information 3 [file peerj-10-13204-s003.docx]

**Information for reviewers:** This questionnaire was sent by mail to the experts, in a link that facilitated its answer and delivery ( <https://forms.gle/idcATxnqToHLRtPt9>) . It has been translated and put in word format, for your disposal.

**Questionnaire for the assessment of the dual-task exercise program model for the improvement of balance and gait speed in the elderly.**

In the following questionnaire, the exercises of the dual-task exercise program to improve balance and gait speed in the elderly will be assessed separately.

The proposed Instrument consists of 11 exercises, divided into 5 levels. Each of the exercises proposed has previously shown its effectiveness in treating factors related to balance and gait, and these exercises are complementary to each other and easy to apply.

We will begin with the first levels in a fixed prioritization training, through which the attention is distributed equally between both tasks, but we aim to progress to a variable prioritization training during levels 4 and 5, so the proportion of Attention directed to the gait and the added task will vary according to the instructions from repeat to repeat.

In assessing each exercise, aspects of clarity of the exercise and suitability for the target population must be taken into account. This assessment is carried out on a Likert-type scale from 1 to 7, the values given to each point being the following:

7= Totally agree

6= Strongly agree

5= Agree

4= Neither agree nor disagree

3= Disagree

2= Strongly disagree

1= Totally disagree

Under each question, there is a box for suggestions or comments, it is requested that if the evaluation is 1-4, leave a comment in which we can analyze the reasons why this exercise may not be appropriate.

## **LEVEL 1 – DUAL TASK SITTING**

Start at this level, with all participants, and depending on their risk or imbalance it will stop at the next level.

**Exercise 1 - Double cognitive-motor task in sitting position**

Position: The older adult should be seated in a standard dining chair.

Exercise: the user must lift their leg up and down alternating between the right and left leg as quickly as possible. The minimum lifting height will be to lift the plantar surface off the ground. They will be carried out in six series of 15 seconds.

Simultaneously with the motor activity, you will be asked for a verbal fluency task, which consists of naming words within a category (example: words that begin with “A”, female names that begin with “M”, names of animals, etc) . The verbal fluency task should be changed in each session.


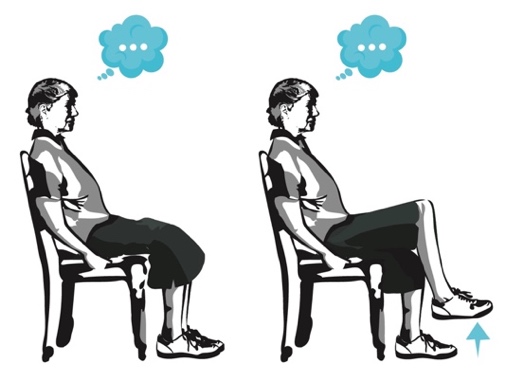


**Mark your level according to the exercise:**

| **Totally disagree**  **1** | **Strongly disagree**  **2** | **Disagree**  **3** | **Neither agree nor disagree**  **4** | **Agree**  **5** | **Strongly agree**  **6** | **Totally agree**  **7** |
| --- | --- | --- | --- | --- | --- | --- |

| **Do you have suggestions for this exercise?** |
| --- |

## **LEVEL 2 – BALANCE IN DUAL TASK**

For this level it is essential that the user can remain standing without the need for external support or help. This cycle is made up of 3 exercises, which are carried out while standing.

***Exercise 2- DT - on stable surface***

The older adult on a firm surface with eyes open, will use a narrow base (feet together) or a tandem posture (depending on the security demonstrated by the participant) that he/she will have to maintain for as long as possible, while performing a cognitive task that they will be able to be, in order of difficulty, randomly name numbers from 100 to 500, association of words by easy categories (animals, fruits, etc.) and more difficult continuums (names of women that begin with a specific letter, cities, etc.). The difficulty of the cognitive task will depend on the safety of the participant’s standing.
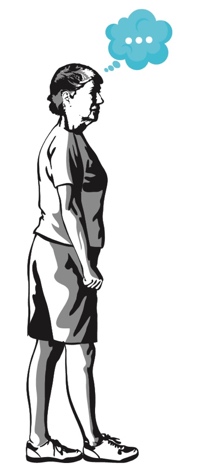


**Mark your level according to the exercise:**

| **Totally disagree** | **Strongly disagree** | **Disagree** | **Neither agree nor disagree** | **Agree** | **Strongly agree** | **Totally agree** |
| --- | --- | --- | --- | --- | --- | --- |

| **Do you have suggestions for this exercise?** |
| --- |

**Exercise 3- Dual motor task on unstable surface.**

On an unstable surface (yoga pad, foam pad), the subject must manipulate an object such as holding a ball or a container. For greater difficulty the subject must grasp or throw the object.


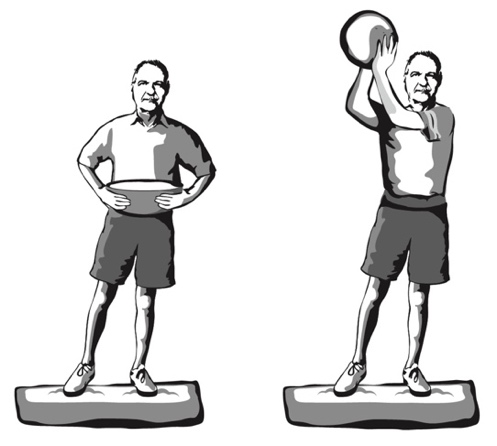


**Mark your level according to the exercise:**

| **Totally disagree** | **Strongly disagree** | **Disagree** | **Neither agree nor disagree** | **Agree** | **Strongly agree** | **Totally agree** |
| --- | --- | --- | --- | --- | --- | --- |

| **Do you have suggestions for this exercise?** |
| --- |

**Exercise 4 - Dual motor cognitive task on unstable surface.**

On an unstable foam surface, with eyes open or closed depending on the user’s safety, with a narrow base or tandem posture, you will perform cognitive activities that will consist of randomly naming odd or even numbers in a certain range, word association (easy and more difficult), or spelling short words backwards.
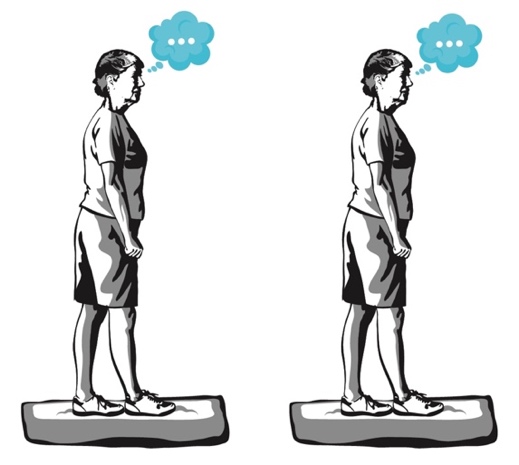


**Mark your level according to the exercise:**

| **Totally disagree** | **Strongly disagree** | **Disagree** | **Neither agree nor disagree** | **Agree** | **Strongly agree** | **Totally agree** |
| --- | --- | --- | --- | --- | --- | --- |

| **Do you have suggestions for this exercise?** |
| --- |

## **LEVEL 3 – TRAINING GAIT IN DUAL MOTOR TASK**

Body displacement work plus manipulation of an object. For this stage it is necessary that the user can move without using external aids such as canes or walkers. In this phase, the two exercises below are used.

**Exercise 5 - Dual motor task on the gait.**

Walking forward, along with a manipulative task, such as walking while lifting an umbrella with both hands, shaking a rattle, while carrying a tray, walking while bouncing a ball.


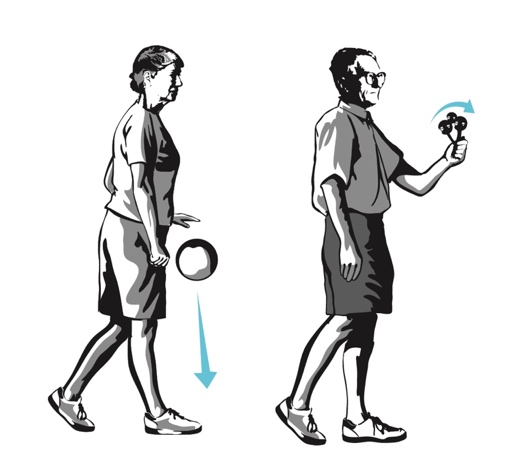


**Mark your level according to the exercise:**

| **Totally disagree** | **Strongly disagree** | **Disagree** | **Neither agree nor disagree** | **Agree** | **Strongly agree** | **Totally agree** |
| --- | --- | --- | --- | --- | --- | --- |

| **Do you have suggestions for this exercise?** |
| --- |

**Exercise 6 - Dual motor task on the gait.**

The older adult must make a movement with lateral steps, with manipulative tasks such as those of the previous exercise added to them.


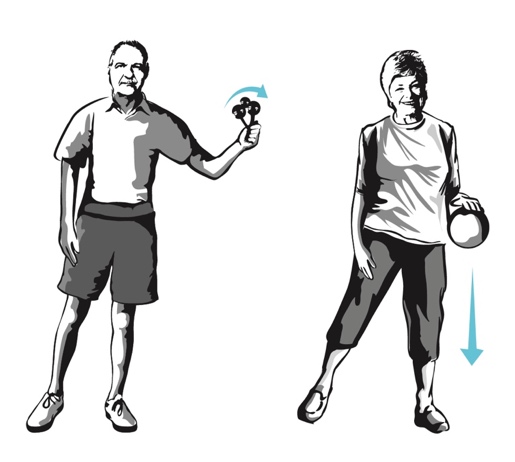


**Mark your level according to the exercise:**

| **Totally disagree** | **Strongly disagree** | **Disagree** | **Neither agree nor disagree** | **Agree** | **Strongly agree** | **Totally agree** |
| --- | --- | --- | --- | --- | --- | --- |

| **Do you have suggestions for this exercise?** |
| --- |

## **LEVEL 4 – TRAINING GAIT IN DUAL TASK**

In this level, body displacement plus cognitive activity are worked on, introducing the use of variable prioritization.

The cognitive activities added to each form of displacement that will be offered will be within the five categories of activities that have been shown to have gait interference in older people:

1. Verbal fluency (for example, cities beginning with a, b, c; names of pets, etc.)
2. Discrimination and decision-making tasks (say yes when you hear dog, but don’t say anything when you hear any other animal)
3. Working memory tasks (subtracts from 100, first in even numbers and the in odd numbers)
4. Mental monitoring tasks (count how many times you hear the “word” in the following story), and
5. Reaction time tasks (react as fast as you can by clapping your hands at the sound of the whistle)

**Exercise 7 – Dual Task gait**

Forward shifts + cognitive activity.


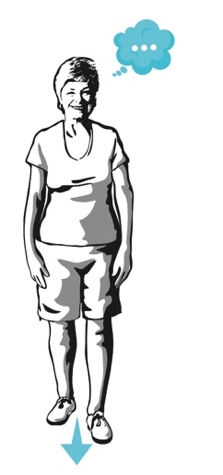


**Mark your level according to the exercise:**

| **Totally disagree** | **Strongly disagree** | **Disagree** | **Neither agree nor disagree** | **Agree** | **Strongly agree** | **Totally agree** |
| --- | --- | --- | --- | --- | --- | --- |

| **Do you have suggestions for this exercise?** |
| --- |

**Exercise 8 - Dual task lateral gait.**

Lateral displacement + cognitive activity.


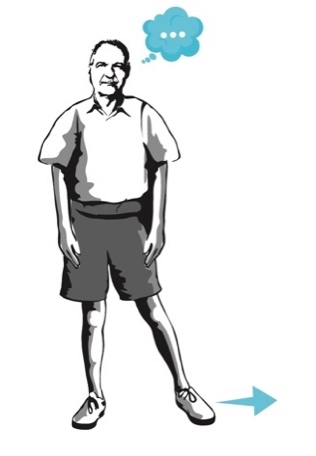


**Mark your level according to the exercise:**

| **Totally disagree** | **Strongly disagree** | **Disagree** | **Neither agree nor disagree** | **Agree** | **Strongly agree** | **Totally agree** |
| --- | --- | --- | --- | --- | --- | --- |

| **Do you have suggestions for this exercise?** |
| --- |

**Exercise 9- Dual task backward gait**

Backward movement + cognitive activity.


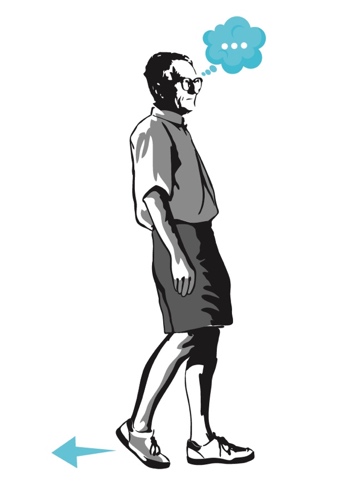


**Mark your level according to the exercise:**

| **Totally disagree** | **Strongly disagree** | **Disagree** | **Neither agree nor disagree** | **Agree** | **Strongly agree** | **Totally agree** |
| --- | --- | --- | --- | --- | --- | --- |

| **Do you have suggestions for this exercise?** |
| --- |

## **LEVEL 5 – DUAL TASK WORK IN DAILY ACTIVITIES.**

During this stage, we seek to carry out two exercises to transfer the dual task to daily activities, and we will continue to make use of variable prioritization.

**Exercise 10 – Gait**

Gait forward avoiding obstacles, while performing a cognitive activity of auditory or visual discrimination.


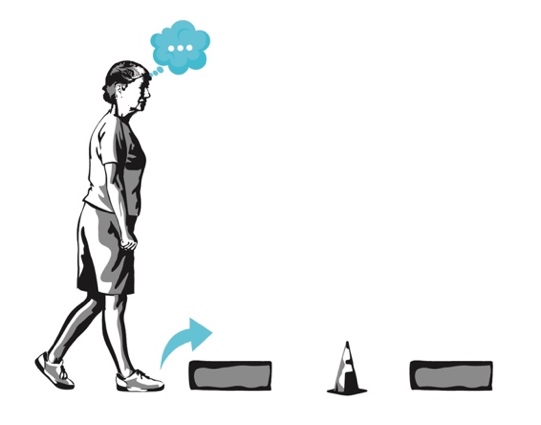


**Mark your level according to the exercise:**

| **Totally disagree** | **Strongly disagree** | **Disagree** | **Neither agree nor disagree** | **Agree** | **Strongly agree** | **Totally agree** |
| --- | --- | --- | --- | --- | --- | --- |

| **Do you have suggestions for this exercise?** |
| --- |

**Exercise 11 – Gait**

Gait forward and twist the trunk to one side or the other according to auditory discrimination (when you hear the whistle, turn to the right; when you hear a palm turn to the left).


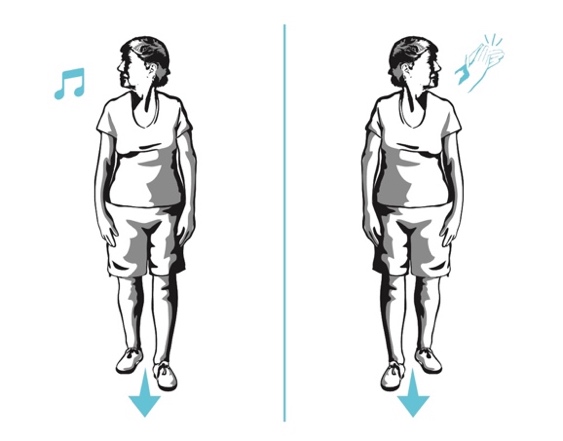


**Mark your level according to the exercise:**

| **Totally disagree** | **Strongly disagree** | **Disagree** | **Neither agree nor disagree** | **Agree** | **Strongly agree** | **Totally agree** |
| --- | --- | --- | --- | --- | --- | --- |

| **Do you have suggestions for this exercise?** |
| --- |

**Thank you for your collaboration in this study.**
